# Influence of Different Time and Frequency of Multienzyme Application on the Efficiency of Broiler Chicken Rearing and Some Selected Metabolic Indicators

**DOI:** 10.3390/ani10030450

**Published:** 2020-03-08

**Authors:** Youssef A. Attia, Mohammed A. Al-Harthi, Ali S. El-Shafey

**Affiliations:** 1 Arid Land Agriculture Department, Faculty of Meteorology, Environment and Arid Land Agriculture, King Abdulaziz University, Jeddah 21589, Saudi Arabia; 2 Animal and Poultry Production Department, Faculty of Agriculture, Damanhour University, Damanhour 22516, Egypt; alielshafey2015@gmail.com

**Keywords:** broilers, application time and way, digestibility, carcass traits, metabolic profiles

## Abstract

**Simple Summary:**

Enzymes are a useful, valuable and economic tool to improve feed utilization and thus animal performance. Costs of enzymes supplementation and time and application frequency of enzymes have received little attention other than dose and type of enzymes. This study showed that multienzymes offered intermittently during both early and late growth periods up to market age of broilers enhanced productive performance and economic profits and can substitute the daily administration with a considerable lowering of the supplementation cost.

**Abstract:**

This study looks at the influence of time and/or frequency of multienzymes application on productivity, carcass characteristics, metabolic profile, and red blood cell characteristics of broiler chickens. Two hundred and eighty, one-day-old Arbor Acres broiler male chicks were randomly distributed into seven treatment groups. Each group consisted of eight replicates of five unsexed birds. The same basal diet was fed in a crumble form to all experimental groups: group one was the unsupplemented control that did not receive multienzymes supplementation. Additionally, multienzymes in water were supplemented in six groups in a factorial arrangement, including three times of application (starter time only which included days 1–21 of age, grower time only which included days 22–37 of age, and starter and grower time which included days 1–37 of age) and two application frequencies (continuously or intermittently). In the continuous application, the multienzymes were added to water over 24 h in a day, while in the intermittent frequency multienzymes were added to water for one day followed by a day off according to the time of application. Regardless of time and frequency of application, enzymes supplementation significantly increased growth rate, feed intake, European Production Index (EPI), protein digestibility, serum albumin, and high-density lipoprotein (HDL). Intermittent multienzymes application during days 1–21 of age or days 22–37 of age resulted in significantly greater growth, better feed conversion rate (FCR), and higher EPI of broilers during the whole rearing period than those under continuous multienzymes during different growth periods. Besides, intermittent multienzymes addition during days 1–37 of age improved FCR of broiler chicks compared to constant application. The intermittent addition of multienzymes during days 1–21 of age or 22–37 days of age and days 1–37 of age caused a significant increase in dry matter (DM) digestibility than the continuous application. The intermittent addition of multienzymes during days 1–21 of age significantly increased the digestibility of crude protein (CP), ether extract (EE), and crude fiber (CF) compared to continuous application. A similar trend was shown in the digestibility of CP and EE due to intermittent use during days 22–37 of age. Intermittent enzymes addition significantly increased high density lipoprotein (HDL) of groups receiving enzymes during days 22–37 of age compared to continuous application of enzymes. In conclusion, the use of multienzymes intermittently during days 1–21 of age and 22–37 days of age significantly increased growth, improved FCR, and raised EPI. Intermittent use can replace continuous multienzyme applications which can save 68.6% of the cost, even though further research is need from the cost-saving edge.

## 1. Introduction

The digestive tract of chickens develops over time and is nearly mature by the fourth week of age, hence the digestibility of nutrients is weak and feed utilization is inadequate during this early age, resulting in increased costs of feeding and environmental pollution [1,2,3,4]. Nutrient digestibility and use of feeds were shown to improve over time and by enzyme supplementations [5,6]. Supplementation with enzymes in broiler diets increased the activity of the digestive enzymes [7,8] and improved the endogenous enzyme production, thus increasing the quantity of nutrients available for absorption [8,9,10]. Nonetheless, multienzymes impacts dietary composition, enzymes type, concentration and profiles, and strain and age of birds [11,12,13,14]. It is clear from the literature that the efficacy of enzymes decreased with the increasing age of chickens, and thus it is correlated with maturation of gut in terms of capacity, endogenous enzyme secretion, and ecology [3,12,13]. It was found that enzymes supplementation had more pronounced effects on broiler chickens than laying hens [2,14] and during the early stage of growth than later ones. The enzyme supplementation is effective for the total feeding cost because costs can be cut considerably if the efficiency of multienzymes can be determined according to the time of feeding [15]. Even so, until now and to the best of our knowledge, no studies that have addressed the relationship between time of growth and time and frequency of multienzymes administration on chickens’ performance, nutrient digestibility, carcass traits, metabolic parameters, and blood constituents are available in the literature. Hence, the hypothesis of this research assumes that multienzymes improve the growth performance of broilers, and the intermittent application during the early growth period may replace the continuous use without adverse effects on growth performance, carcass traits and metabolic profits while enhancing economic profits. 

## 2. Materials and Methods 

### 2.1. Animals and Dietary Treatments

Two hundred and eighty, one-day-old Arbor Acres broiler chicks were randomly distributed into seven treatment groups. Each group consisted of eight replicates of five male chicks each. The same basal diet was offered in crumble form to all experimental groups. The group one (unsupplemented control group) was fed an un-supplemented diet. Further, there were six multienzyme-supplemented groups in a factorial arrangement that included three groups supplemented during different times of growth by two frequencies of administration. The times of growth were: the starter time only (days 1–21 of age), the grower time only (days 22–37 of age), and the starter plus grower time (days 1–37 of age). The administration of the multienzymes within each time of application were continuous or intermittent. In the continuous frequency, the multienzymes were added to the water over 24 h in a day. In the intermittent frequency, the multienzymes were added to the water for one day, followed by a day off. The diets in this experiment were formulated to meet the requirements of broiler chickens [16]. 

The multienzymes (Galzym® produced by Tex Biosciences (P) limited, Ashok Nagar, Chennai, India) used were a combination of a group of exogenous and fibrolytic enzymes consisting of: cellulase (100,000,000 units), xylanase (1,500,000 units), lipase (6500 units), alpha amylase (25,0000 units), protease (40,0000 units) and Pectinase (30,000 units). The recommended dose of enzymes was 0.333 mL/1 L water. The composition of the experimental diets is presented in Table 1.

All the experimental procedures were approved by Deanship of Scientific Research King Abdulaziz University, under proposal number G 170-155-1440H. The treatment of animals was according to the Royal Decree number M59 in 14/9/1431H, which recommends animal rights, welfare, and minimal stress and prevents any harm or suffering.

### 2.2. Animal Housing and Management

Broilers were housed in batteries of five chickens per cage (30 × 35 × 45 cm). Chicks were offered free access to feed and water during the experimental period. The housing temperature was 33 °C during the 1st week and declined gradually by 2 °C each week and was kept constant at 25 °C until slaughter. The average minimum and maximum outdoor and indoor relative humidity and temperature were 25.7 and 38.6%, 48.5 and 54.2%, 28.7 and 37.5 °C, and 27.8 and 34.6 °C, respectively. The light-dark cycle schedule was 23:1 h daily. 

### 2.3. Experimental Measurements 

Broilers in each replicate were weighed (g) at day 1, 21, and 37 of age, and the body weight gain (BWG) (g/chick) was calculated. Feed intake was recorded for each replicate (g/chick), and thereby FCR (g feed/g gain), and survival rate (SR, 100 − mortality rate) were calculated during days 1–21, 22–37, and 1–37 of age. Water intake was not measured due to a lack of precise research facility. European production index was for the index of economic evaluation, as reported by [12]. 

Apparent digestibility for crude protein (CP), dry matter (DM), ether extract (EE), crude fiber (CF), nitrogen-free extract (NFE) and ash during different times of growth using various methods of application was determined using Celite^TM^ (Celite Corp., Lompar, CA, USA) as a marker according to Dourado et al. [17]. The total collection method of excreta was followed for 5 days in each application time during days 15–20, 25–30, and 32–36 of age, respectively. In each time (1–21), (22–37) and (1–37) excreta and feed samples were constituted, and samples of each (25%) were collected, cleaned from feed residuals, scale and feathers, and then the DM, CP, EE, CF and ash of feeds and excreta were assayed according to [18] and expressed on dry matter basis. The NFE of feed and excreta was estimated by differences between the dry matter and sum of CP, EE, CF, and ash. The method adopted by [17] was used for analyses of acid-insoluble ash (AIA). The AIA recovery was estimated as g execrated/g intake × 100 using dry matter basis of excreta voided and feed intake data. It was found to range from 94% to 109%.

At 37 days of age, eight male broiler chicks per treatment group were slaughtered after 8 h fasting according to the Islamic method using a sharp knife and cutting into the jugular vein, carotid artery and windpipe, processed, and the weight of the carcass and inner organs was measured and estimated as a percentage of live body weight.

At 37 days of age, eight blood samples of 5 mL each per treatment group were collected and equally divided into two clean tubes with or without heparin. The tubes without heparin were centrifuged at 1500× *g* for 10 min at 4 °C, and then serum was separated and stored at −18 °C until analysis. The serum profiles were determined using commercial diagnostic kits (Diamond Diagnostics Company, Cairo, Egypt). Total protein (g/dl) [19], albumin (g/dl) [20], and globulin (g/dl) [21] were estimated. In addition, triglycerides [22], total cholesterol [23], high-density lipoprotein (HDL) [24]; and low-density lipoprotein (LDL) [25] were appraised. 

Red blood cells [26], hemoglobin [27], packed cells volume (PCV) [28], mean cell volume (MCV), mean cell hemoglobin (MCH), and mean cell hemoglobin concentration (MCHC) [29] were manually measured.

### 2.4. Statistical Analysis

The statistical analysis software (SAS) was done using a completely randomized design and data were subjected to a two-way ANOVA procedure based on only time of supplementation and administration frequency without the unsupplemented control [30]. The statistical model included the effects of the time of multienzyme application (starter, grower-finisher and whole period), frequency of application (continuously vs. intermittently) and their interactions according to the subsequent model:

Yijk = μ + Di + AMj + (D × AM)+ eijk(1)

where: Y = the dependent variables; μ = general mean; D = time effect; AM = administration frequency effect; (D × AM) = interaction between the two factors; e = random error. Orthogonal contrast was done to test the effect of enzyme treatment regardless of time and frequency of application against the unsupplemented control. Before analysis, all percentages were subjected to logarithmic transformation (log10x+1) to normalize data distribution. The mean differences were determined using student Newman Keuls [30]. When *p* values found to be between 0.05 and 0.1 were considered as a tendency and thus indicate in the results “tend to be higher/lower.”

## 3. Results

The effect of different times of multienzymes application in water continuously or intermittently on the production performance of broiler chickens is summarized in Table 2. 

The contrast analyses showed that multienzymes supplementation significantly increased the growth of broilers during days 1–21 of age and days 1–37 of age compared to the unsupplemented control. 

Both application time and application frequency had significant effects on the growth of broilers during 1–21, 22–37, and 1–37 days of age, but these effects were confounded by the significant interaction between the time and the frequency of application. 

The interaction between application time and application frequency of multienzymes was significant for 1–21, 22–37, and days 1–37 of age. It was found that intermittent use during days 1–21 of age significantly improved the rate of growth of broilers during periods 1–21, 22–37, and 1–37 days of age compared with other continuous or intermittent groups. Moreover, intermittent enzyme application during days 22–37 of age substantially increased the growth of broilers during periods 1–37 days of age compared to continuous enzyme supplementation during periods 1–21 and 22–37 days of age, and permanent and intermittent use during periods 1–37 days of age. Enzyme application either as constant or intermittent during days 1–37 of age showed a similar growth rate during all tested periods.

The contrast analyses indicated that feed intake was significantly higher for chickens fed enzyme-supplemented diets compared to the un-supplemented ones during periods 1–21 and 1–37 days of age.

Feed intake during periods 1–21, 22–37, and 1–37 days of age was not significantly affected by the time of application and the interaction between time and frequency of enzyme application. However, feed intake during 22–37 and 1–37 days of age was significantly higher for broilers fed diets supplemented with enzymes continuously compared to those on the intermittent addition. 

The contrast analyses for FCR showed no significant difference between the enzyme treatments and the un-supplemented group. The time of application significantly affected FCR during days 1–37 of age and application frequency influenced FCR during different tested periods, but these effects were confounded by the interaction between the time and frequency of application during days 1–21 and 1–37 of age. 

In general, intermittent application during days 1–21 and 22–37 of age significantly improved FCR of broilers compared to continuous supplementation during periods 1–21 and 1–37 days of age. Besides, intermittent enzyme application during 1–37 days of age significantly improved FCR for broilers during days 1–37 compared to constant use.

There was no significant effect of time and/or frequency of enzyme application on the survival rate of broilers during days 1–37 of age. 

The contrast analyses showed that enzyme application increased EPI compared to the unsupplemented control. The time and/or frequency of enzyme application significantly affected EPI. The intermittent enzyme application during days 1–21 of age significantly improved EPI of broilers compared to other groups. In addition, intermittent enzyme application during days 22–37 of age significantly enhanced EPI of broilers compared with other groups on continuous or intermittent frequency, except for intermittent frequency during days 1–21 of age. The intermittent application during 1–21 days of age had a greater effect on EPI than the same use during 22–37 days of age. 

Results concerning the influence of the time and/or application frequency on nutrients digestibility of broilers are shown in Table 3. The contrast analyses demonstrated that enzyme supplementations significantly increased only crude protein digestibility compared to unsupplemented control, and had no effect on other digestibility traits. 

Time and frequency of enzyme application had a significant effect on the digestibility of DM, CP, EE, and CF, but these factors were confounded by the interaction between the time and frequency of enzyme application. The interaction between application time and/or application frequency had a significant impact on most nutrient digestibility with the exception of the digestibility of NFE (*p* = 0.834) and ash (*p* = 0.078).

The interaction effect indicates that the intermittent use of enzymes during 1–21, 22–37, and days 1–37 of age improved the DM digestibility compared with continuous applications.

The intermittent application of multienzymes during days 1–21 of age improved the digestibility of CP, EE, and CF compared to the other groups. The intermittent application of enzymes during days 22–37 of age enhanced the digestibility of CP and EE compared with the continuous and intermittent use during days 1–37 of age. The intermittent application during 1–21 days of age increased the digestibility of DM, CP, EE, and CF more than intermittent use during 22–37 days of age. 

There were no significant differences between continuous and intermittent enzyme applications during days 1–37 of age on the digestibility of CP, EE, and CF. 

Regardless of the time of application, intermittent supplementation increased ash digestibility compared to continuous supplementation. 

The carcass characteristics and the inner organs of the broilers influenced by time of multienzymes application and/or the way of addition are shown in Table 4. The contrast analyses showed no significant effect of multienzymes compared to the unsupplemented control on all carcass traits and inner body organs. 

Dressing, abdominal fat, gizzard, proventriculus, intestinal tract, liver, and heart were not significantly affected by the time and frequency, or by the interaction between time and frequency of application of enzymes. 

The pancreas percentage was affected (*p* = 0.051) by the time of application, showing a trend of high value with enzymes supplemented during days 22–37 of age compared to the other periods. The effect of the application frequency approached significant (*p* = 0.092) for the heart percentage, showing the increase was proportional due to intermittent use. 

The biochemical indices of blood serum of the broilers affected by the time of application and/or the application frequency are shown in Table 5.

The contrast analyses showed that enzyme treatments significantly increased the albumin, albumin/globulin ratio, total lipids, HDL, and HDL/LDL ratio in blood serum compared to the unsupplemented control, but decreased serum globulin. Differences between the enzymes’ treatment and the unsupplemented control approached significant for total protein (*p* < 0.054).

There was no significant impact of time of application and/or frequency of use on blood serum proteins (total protein, albumin, globulin, albumin/globulin ratio). On the other hand, plasma total lipids were significantly affected by the application time, showing that total lipids were lower when enzymes were applied during days 22–37 of age than during the other times. The effect of time of application approached significant (*p* < 0.063) for serum triglycerides, with the highest being the 1–21 days group.

There was a significant interaction between application time and application frequency for serum HDL, LDL, and LDL/HDL ratio. Enzyme application intermittently during days 1–21 of age significantly decreased serum LDL compared to other groups, except for the continuous use during days 1–37 of age. On the other hand, the continuous offer of multienzymes during days 1–21 and 1–37 of age significantly increased the HDL and HDL/LDL ratio compared to the intermittent application, but the contrary was observed during days 22–37 of age.

Characteristics of red blood cells of broiler chickens are reported in Table 6. There were no significant effects due to enzyme treatments compared to the unsupplemented control when contrast analyses were considered for all red blood parameters. 

The application time, application frequency, and the interaction between time and frequency of enzyme application did not significantly affect red blood cell traits, except for a significant increase in MCHC (*p* < 0.039) due to the continuous use compared to intermittent addition. 

## 4. Discussion

It is interesting to report that enzyme supplementation regardless of time and frequency of application enhanced growth performance and protein digestibility compared to unsupplemented controls. The increase in the growth of enzyme-supplemented groups was connected with increasing feed intake and digestibility of crude protein. These results are similar to the findings by [8,12,31,32,33,34]. The effect of the enzyme cocktail was attributed to improved digestibility and increased protein and energy availability for growth [32,33,34,35,36,37,38,39,40].

The intermittent enzymes application during days 1–21 of age resulted in better growth, improved FCR, and improved EPI compared to the continuous administration along the same period, but also to the constant or intermittent use during the other tested times (22–37 and days 1–37 of age). The positive effects of offering enzymes during the early growth phase (1–21 days of age) may be due to the supportive influence of exogenous enzymes on gut development and functions [3,13,15], and thus enhancing the digestibility of nutrients during the first stage of growth [1,10]. The gut of young chicks is under development and maturation in terms of enzyme secretion, capacity, and ecology during day 1–28 of age [1,5]. 

The results demonstrated that intermittent administration was adequate, which can save considerable cost (68.6%) of enzyme application. The high growth and better FCR of broilers on the intermittent use condition are tied to the increased digestibility of DM, CP, EE, and CF. Similar results concerning the positive impact of enzymes on nutrient digestibility were recently cited [6,8,13,31]. 

However, continuous use of enzymes throughout the experimental period (1–37 days of age) neither improved growth performance nor the digestibility of nutrients, and this may be due to the negative feedback mechanism of exogenous enzymes on pancreatic enzyme secretion [12,15].

It was found that the application of multienzymes during the period from 1 to 21 days of age yielded stronger effects than the use during the later ages and the whole period of growth on BWG and FCR than those supplemented during 22–37 or and days 1–37 of age. This may be because the digestive tract is under development up to four weeks of age. The enzyme applications during days 1–37 of age yield the least response. The continuous use during days 22–37 of age produced better growth during days 1–37 of age and FCR during days 1–21 and 1–37 of age than other permanent groups. The relationship between the time of application and growth performance of broilers needs, therefore, further research. 

The positive impact of enzymes on animal productivity reported herein is in concert with those observed by [7,14,32]. These enhancements were attributed to the increasing activity of digestive enzymes [7,13], as estimated by indirect measurements, such as growth performance, or directly by digestibility of nutrients and eliminating the adverse effects of anti-nutritional factors [6,33,34]. Besides, multienzymes were found to increase the use of energy in sorghum and maize-soybean meal-based diets due to enhancing cereal cell walls and starch digestion [3,10]. Enzyme application may improve beneficial intestinal microbiota favoring *Lactobacillus* [14,31] and reduce environmental pollution [6,15] due to increased digestibility of nutrients (protein, fat and fiber, and minerals), while decreasing nutrients available in the hindgut for growth of harmful microbiota and increasing animal productive performance [9,13,15]. However, multienzymes’ influence depends on enzyme type and concentrations, diet profile, form [4,6,31,35], and stage of growth [11,15].

Based on the present results, an enzyme blend supplemented in water during a different time of growth by various application frequencies at the recommended level did not affect most of the carcass and organ traits of broiler chickens. However, the pancreas percentage was increased due to enzyme supplementations during days 22–37 of age, suggesting a negative feedback mechanism of exogenous enzymes on the pancreas function [2,36]. There was also a decrease in proventriculus percentage when enzymes were added during the early time (1–21 days of age) and the growing time (22–37 days of age). In general, the absence of effects of enzymes on most of carcass and organ traits is constant with the findings by [7,37,38,39]. These authors indicated that application with enzymes did not affect carcass parameters and body organs of broilers and Japanese quail except for liver percent that was reduced with multienzymes application. Likewise, carcass traits were not influenced by dietary enzyme addition to broiler chicken diets [29,40,41]. 

In general, with few exceptions, our results showed that there were no significant effects from the application time and/or application frequency on the blood serum biochemistry and hematology. In other studies, dietary enzyme addition did not affect serum protein concentrations (alpha 1-, alpha 2-, beta and gamma-globulins and albumin) and albumin/globulin ratios [42,43,44,45]. 

The most pronounced effects found herein were a decrease in plasma cholesterol due to the continuous addition of enzymes and an increase in HDL with the intermittent applications during days 22–37 of age. There was also a decrease in LDL when enzymes were supplemented intermittently during days 1–21 of age. These beneficial effects due to multienzyme application on lipid metabolites require further investigation. However, enzymes had no significant effect on concentrations of triglyceride and cholesterol compared with animals fed on the same diet without the addition of enzymes [46]. Similar to the current results, different blood cholesterols and HDL/LDL ratios were increased in the unsupplemented control groups than enzyme-supplemented groups, showing the positive influence of enzymes on blood cholesterol [7,41,47]. 

## 5. Conclusions

Enzymes positively affect growth rate, feed intake, EPI, protein digestibility, serum albumin, total lipids, and HDL compared to the control group, irrespective of time and frequency of enzyme application. The application of multienzymes intermittently during 1–21 days of age and 22–35 days of age for broilers significantly increased growth, improved FCR, and enhanced EPI. This can replace continuous multienzymes applications while saving 68.6% of the cost, even though further research is needed to explore the beneficial economic impact of of time and frequency of enzyme application. 

## Figures and Tables

**Table 1:** Ingredients and chemical-nutritional composition of the experimental diets (as feed basis) during different experimental periods.

Ingredients (g/kg)	Diets	
	Starter	Grower-Finisher
Maize	502.3	508.1
Rye	0	50
Soybean meal, 44% crude protein	328	244
Dicalcium phosphate	18.00	16.00
Limestone	10.00	10.00
NaCl	3.00	4.50
Full fat soybean meal	100	130
Vit+Min premix ^1^	3.00	3.00
L-Lysine	1.00	1.90
DL-Methionine	2.00	2.50
Vegetable oil	22.70	20.00
Celite ^TM^	10.0	10.0
Calculated and determined composition, (g/kg)		
Dry matter ^2^	891	896
Crude protein, % ^2^	223	205
Metabolisable energy (MJ/kg) ^3^	12.50	12.64
Crude fat,% ^2^	58	61
Crude fibre,% ^2^	34.1	33.3
Nitrogen free extract ^2^	513.4	532
Calcium ^3^	8.72	8.13
Available phosphorus ^3^	4.17	3.88
Methionine ^3^	5.42	5.68
Methionine+cystine ^3^	9.10	9.05
Lysine ^3^	13.08	12.41
Ash ^2^	62.5	64.7

^1^ Vit+Min mix. provides per kilogram of the diets: Vit. A, 12,000 internation unit (IU), vit. E (dl-α-tocopheryl acetate) 20 mg, menadione 2.3 mg, Vit. D_3_, 2200 international chick unit (ICU), riboflavin 5.5 mg, calcium pantothenate 12 mg, nicotinic acid 50 mg, Choline 250 mg, vit. B_12_ 10 µg, vit. B_6_ 3 mg, thiamine 3 mg, folic acid 1 mg, d-biotin 0.05 mg. Trace mineral (mg/kg of diets): Mn 80 Zn 60, Fe 35, Cu 8, Selenium 0.1 mg. ^2^ Analyzed values. ^3^ Calculated values.

**Table 2:** Effect of multienzymes supplemented in water continuously or intermittently during the starter, grower, and the whole time on growth performance of broiler chicks.

Treatments/Period	Body Weight Gain (g)			Feed Intake (g)			Feed Conversion Rate (g/g)			Survival Rate (%)	Production Index
	1–21 d	22–37 d	1–37 d	1–21 d	22–37 d	1–37 d	1–21 d	22–37 d	1–37 d		
Effect of enzyme treatments vs unsupplemented control (contrast)											
Control	573 ^b^	1113	1686 ^b^	907 ^b^	2119	3026 ^b^	1.59	1.91	1.80	97.5	246 ^b^
Enzymes	674 ^a^	1139	1813 ^a^	1031 ^a^	2093	3125 ^a^	1.53	1.84	1.72	99.6	286 ^a^
Effect of administration time											
1–21	692 ^a^	1158 ^a^	1850 ^a^	1031	2066	3096	1.50	1.79	1.68 ^b^	100	301 ^a^
22–37	676 ^b^	1156 ^a^	1831 ^a^	1020	2106	3126	1.51	1.82	1.71 ^ab^	98.8	287 ^b^
1–37	654 ^c^	1102 ^b^	1755 ^b^	1043	2108	3152	1.57	1.90	1.77 ^a^	100	269 ^c^
Effect of administration frequency											
Continuous	658 ^b^	1117 ^b^	1775 ^b^	1035	2128 ^a^	3164 ^a^	1.58 ^a^	1.91 ^a^	1.78 ^a^	99.2	267 ^b^
Intermittent	690 ^a^	1160 ^a^	1850 ^a^	1027	2058 ^b^	3086 ^b^	1.47 ^b^	1.77 ^b^	1.66 ^b^	100	304 ^a^
Interaction between time and frequency of application											
1–21 Con	653 ^c^	1093 ^d^	1746 ^d^	1047	2107	3154	1.60 ^a^	1.93	1.81 ^a^	100	261 ^c^
1–21 Int	731 ^a^	1222 ^a^	1953 ^a^	1014	2024	3038	1.39 ^e^	1.66	1.56 ^d^	100	340 ^a^
22–37 Con	667 ^bc^	1143 ^bc^	1810 ^c^	1027	2143	3170	1.54 ^c^	1.88	1.75 ^b^	97.5	273 ^c^
22–37 Int	685 ^b^	1167 ^b^	1852 ^b^	1012	2069	3082	1.48 ^d^	1.77	1.66 ^c^	100	300 ^b^
1–37 Con	652 ^c^	1114 ^cd^	1766 ^d^	1031	2135	3167	1.58 ^ab^	1.92	1.79 ^a^	100	267 ^c^
1–37 Int	654 ^c^	1089 ^d^	1743 ^d^	1054	2081	3136	1.55 ^bc^	1.88	1.75 ^b^	100	270 ^c^
RMSE	32.9	41.3	37.8	52.4	84.9	88.5	0.071	0.109	0.067	5.21	13.5
*p* value											
Control vs. enzymes	0.002	0.609	0.011	0.001	0.975	0.012	0.620	0.672	0.750	0.465	0.001
Time	0.048	0.009	0.001	0.616	0.462	0.391	0.088	0.112	0.018	0.552	0.001
Frequency	0.012	0.009	0.001	0.679	0.034	0.024	0.001	0.002	0.001	0.693	0.001
Interaction	0.041	0.001	0.001	0.492	0.927	0.562	0.017	0.066	0.005	0.348	0.001

D = day; Con = Continuous; Int = Intermittent; RMSE = Root mean square error; a–d, means with different superscripts in the same column in similar treatment groups are significantly different.

**Table 3:** Effect of multienzymes supplemented in water continuously or intermittently on apparent nutrient digestibility of broiler chicks.

Treatments/Period	Apparent Nutrient Digestibility, %					
	Dry Matter	Crude Protein	Ether Extract	Crude Fiber	Nitrogen Free Extract	Ash
Unsupplemented control vs. enzymes treatment (contrast)						
Control	83.2	74.4 ^b^	85.2	27.3	76.9	34.8
Enzymes	85.6	79.5 ^a^	88.4	29.7	77.6	37.3
Effect of administration time						
1–21	86.8 ^a^	80.5 ^a^	89.6 ^a^	30.2 ^a^	78.0	37.9
22–37	85.0 ^b^	79.5 ^b^	87.9 ^b^	29.8 ^a^	77.3	37.0
1–37	84.9 ^b^	78.5 ^c^	87.6 ^b^	28.9 ^b^	77.3	36.9
Effect of administration frequency						
Continuous	84.2 ^b^	78.5 ^b^	87.2 ^b^	28.8 ^b^	77.1^b^	36.8 ^b^
Intermittent	86.9 ^a^	80.5 ^a^	89.5 ^a^	30.5 ^a^	78.0^a^	37.8 ^a^
Interaction between time and frequency of application						
1–21 Con	83.9 ^c^	78.5 ^c^	86.9 ^d^	28.3 ^e^	76.8	36.7 ^b^
1–21 Int	89.7 ^a^	82.5 ^a^	92.3 ^a^	32.1 ^a^	77.9	39.2 ^a^
22–37 Con	84.3 ^c^	78.7 ^c^	87.1 ^cd^	29.5 ^bc^	77.4	36.6 ^b^
22–37 Int	85.5 ^b^	80.2 ^b^	88.7 ^b^	30.1 ^b^	78.6	37.1 ^b^
1–37 Con	84.3 ^c^	78.2 ^c^	87.6 ^cd^	28.6 ^de^	77.1	36.9 ^b^
1–37 Int	85.7 ^b^	78.8 ^c^	87.7 ^c^	29.3 ^cd^	77.5	37.1 ^b^
RMSE	2.61	1.76	2.74	1.52	2.42	1.74
*p* value						
Control vs. enzymes	0.732	0.001	0.554	0.866	0.399	0.271
Time	0.045	0.002	0.049	0.036	0.565	0.116
Fequency	0.001	0.001	0.001	0.001	0.157	0.027
Interaction	0.011	0.011	0.011	0.002	0.834	0.078

Con = Continuous; Int = Intermittent; RMSE = Root means square error; a–e, means with different superscripts in the same column in similar treatment groups are significantly different.

**Table 4:** Effect of multienzymes supplemented in water continuously or intermittently during the starter, grower, and the whole times on carcass characteristics and inner body organs of 37-day-old broiler chicks.

Treatments/Period	Dressing and Inner Organs,%							
	Dressing	Abdominal Fat	Gizzard	Proventriculus	Intestinal	Liver	Pancreas	Heart
Unsupplemented control vs enzymes treatment (contrast)								
Control	74.7	0.983	1.23	0.333	4.83	2.23	0.235	0.418
Enzymes	71.7	1.12	1.17	0.387	5.08	2.04	0.190	0.457
Effect of administration time								
1–21	74.1	1.078	1.16	0.379	5.12	2.14	0.186	0.434
22–37	70.5	1.170	1.21	0.388	5.15	2.10	0.213	0.476
1–37	70.3	1.140	1.12	0.394	4.96	1.88	0.171	0.461
Effect of administration frequency								
Continuous	71.6	1.199	1.15	0.411	5.04	2.07	0.193	0.439
Intermittent	71.7	1.054	1.18	0.363	5.11	2.01	0.187	0.475
Interaction between time and frequency of application								
1–21 Con	73.1	1.058	1.07	0.402	5.06	2.18	0.182	0.414
1–21 Int	75.1	1.098	1.25	0.356	5.18	2.10	0.190	0.454
22–37 Con	71.9	1.190	1.20	0.372	5.08	2.10	0.230	0.454
22–37 Int	68.7	1.150	1.22	0.404	5.22	2.10	0.196	0.498
1–37 Con	69.7	1.350	1.17	0.458	4.98	1.92	0.168	0.450
1–37 Int	71.3	0.877	1.07	0.330	4.94	1.84	0.174	0.472
RMSE	4.41	0.329	0.169	0.078	1.06	0.292	0.036	0.055
*p* value								
Control vs. enzymes	0.233	0.174	0.333	0.268	0.906	0.172	0.553	0.311
Time	0.122	0.821	0.510	0.911	0.911	0.122	0.051	0.244
Frequency	0.938	0.222	0.625	0.110	0.851	0.622	0.624	0.092
Interaction	0.341	0.219	0.184	0.093	0.979	0.941	0.369	0.893

Con = Continuous; Int = Intermittent; RMSE = Root means square error.

**Table 5:** Effect of multienzymes supplemented in water continuously or intermittently during the starter, grower, and the whole times on blood biochemical constituents of 37-day-old broiler chicks.

Treatments/Period	Blood Biochemical Constituents									
	Total Protein, g/dL	Albumin, g/dL	Globulin, g/dL	A/G Ratio	Total Lipids, mg/dL	Triglycerides, mmol/L	Cholesterol, mmol/L	HDL, mmol/L	LDL, mol/L	LDL/HDL Ratio
Unsupplemented control vs. enzyme treatments (contrast)										
Control	6.38	2.92 ^b^	3.46 ^a^	0.844 ^b^	7.4 ^b^	2.07	5.56	0.936 ^b^	2.23	0.421 ^b^
Enzymes	6.21	2.98 ^a^	3.23 ^b^	0.931 ^a^	9.3 ^a^	1.94	5.73	0.978 ^a^	2.09	0.466 ^a^
Effect of administration time										
1–21	6.24	3.03	3.21	0.953	9.7 ^a^	1.95	5.73	0.993 ^a^	2.12	0.469
22–37	6.17	3.03	3.15	0.975	8.5 ^b^	1.92	5.73	0.996^a^	2.09	0.475
1–37	6.21	2.87	3.34	0.864	9.7 ^a^	1.94	5.73	0.944^b^	2.07	0.456
Effect of administration frequency										
Continuous	6.18	2.99	3.18	0.951	9.6	1.93	5.66^b^	0.998^a^	2.12^a^	0.471
Intermittent	6.23	2.96	3.28	0.910	9.0	1.94	5.79^a^	0.957^b^	2.07^b^	0.461
Interaction between time and frequency of application										
1–21 Con	6.26	3.10	3.16	0.984	9.4	1.94	5.66	1.071 ^a^	2.21^a^	0.485 ^a^
1–21 Int	6.22	2.96	3.16	0.923	10.0	1.97	5.81	0.915 ^bc^	2.02^c^	0.453 ^bc^
22–37 Con	6.10	3.08	3.02	1.036	9.4	1.92	5.68	0.946 ^bc^	2.09^b^	0.452 ^bc^
22–37 Int	6.24	2.98	3.28	0.915	7.6	1.92	5.78	1.045 ^a^	2.10^b^	0.497 ^a^
1–37 Con	6.18	2.80	3.38	0.834	10.0	1.92	5.66	0.978 ^b^	2.05^bc^	0.476 ^b^
1–37 Int	6.24	2.94	3.30	0.893	9.4	1.95	5.80	0.910 ^c^	2.10^b^	0.434 ^c^
RMSE	0.146	0.206	0.251	0.131	1.13	0.030	0.062	0.047	0.051	0.024
*p* value										
Control vs. enzymes	0.054	0.048	0.009	0.025	0.001	0.731	0.896	0.005	0.351	0.029
Time	0.689	0.156	0.249	0.150	0.039	0.063	0.995	0.035	0.219	0.197
Frequency	0.327	0.661	0.318	0.395	0.159	0.188	0.001	0.023	0.025	0.281
Interaction	0.399	0.279	0.333	0.311	0.081	0.384	0.605	0.001	0.001	0.001

Con = Continuous; Int = Intermittent; HDL = High density lipoprotein; LDL = Low density lipoprotein; RMSE = Root mean square error; a–c, means with different superscripts in the same column in similar treatment groups are significantly different.

**Table 6:** Effect of multienzymes supplemented in water continuously or intermittently during the starter, grower and the complete times on red blood cell characteristics in broiler chicks.

Treatments/Period	Red Blood Cell Characteristics, %					
	RBC’s (106/mm^3^)	Hemoglobin (g/dL)	PCV (mL/1 mL)	MCV, µm^3^/RBC	MHC, pg	MCHC, %
Unsupplemented control vs enzymes (contrast)						
Control	1.52	10.5	32.3	217	70.9	32.9
Enzymes	1.33	11.2	33.2	250	84.0	33.6
Effect of administration time						
1–21	1.34	11.4	33.5	252	84.8	33.7
22–35	1.31	11.1	33.0	253	84.91	33.6
1–35	1.35	11.1	33.1	246	82.47	33.5
Effect of administration frequency						
Continuous	1.31	11.3	33.2	253	86.4	34.1
Intermittent	1.35	11.1	33.2	246	81.6	33.1
Interaction between time and frequency of application						
1–21 Con	1.34	11.6	33.8	253	86.7	34.3
1–21 Int	1.34	11.1	33.2	250	82.9	33.1
22–35 Con	1.26	11.0	32.4	257	87.3	33.9
22–35 Int	1.36	11.2	33.6	248	82.5	33.3
1–35 Con	1.34	11.4	33.4	250	85.4	34.1
1–35 Int	1.36	10.8	32.8	242	79.6	32.9
RMSE	0.089	0.87	1.89	21.1	7.87	1.26
*p* value						
Control vs. enzymes	0.991	0.718	0.741	0.848	0.799	0.862
Time	0..589	0.838	0.824	0.756	0.742	0.948
Frequency	0.232	0.302	0.993	0.370	0.108	0.039
Interaction	0.429	0.501	0.482	0.916	0.959	0.825

Con = Continuous; Int = Intermittent; PCV = Packed cell volume; MCV = Mean cell volume; MCH = Mean hemogloblin concentration; MCHC = Mean cell hemogloblin concentration; RMSE = Root means square error.
